# Tapia's Syndrome: A Comprehensive Analysis of a Rare Intensive Care-Associated Complication

**DOI:** 10.7759/cureus.53486

**Published:** 2024-02-03

**Authors:** Rita Piteira, Diogo Marques, Filipa Carrega, Rita Silvério, Manuela Fera

**Affiliations:** 1 Internal Medicine, Centro Hospitalar de Setúbal, Setúbal, PRT; 2 Neuroradiology, Hospital Garcia de Orta, Almada, PRT

**Keywords:** dysphonia, dysphagia, orotracheal intubation, intensive care, paralysis, recurrent laryngeal nerve, hypoglossal nerve, tapia's syndrome

## Abstract

Tapia's syndrome is a rare complication of airway manipulation, involving the simultaneous paralysis of the hypoglossal nerve and the recurrent laryngeal nerve. The etiological mechanism is commonly attributed to compression or stretching during airway manipulation. An efficient recognition of this condition is pivotal for a comprehensive multidisciplinary approach and optimized recovery time. The presence of persistent dysphagia and dysphonia, coupled with observable deviation or restriction of tongue movement, not only after oral endotracheal intubation for surgical interventions with general anesthesia but also after a prolonged orotracheal intubation period in the intensive care, should heighten the suspicion of this syndrome. This report details a case of Tapia's syndrome emerging as a complication of airway manipulation and prolonged intubation in the intensive care unit.

## Introduction

Tapia's syndrome was first described by the Spanish otolaryngologist Antonio Garcia Tapia in 1904 [1]. This rare condition encompasses two distinct scenarios that manifest with similar clinical characteristics: a central type with an intramedullary lesion affecting the nucleus ambiguus and the pyramidal tract and a peripheral type caused by an extracranial lesion with the involvement of the hypoglossal nerve (XII) and the recurrent laryngeal branch of the vagal nerve (X) at the pyriform fossa, where these two nerves intersect [2]. The peripheral type is more extensively documented in the literature and is characterized by unilateral or bilateral paralysis of the tongue and ipsilateral vocal cord while sparing the soft palate and pharynx motility [3]. Clinical signs and symptoms comprise hoarseness or dysphonia, dysphagia, and the unilateral deviation of the tongue toward the denervated side [4].

## Case presentation

A 34-year-old male patient with a history of type 1 diabetes mellitus and epilepsy was admitted to the emergency department due to diabetic ketoacidosis and seizures resulting from therapeutic non-compliance. Ketoacidosis was fully compensated on the first day, but due to lingering partial seizures, there was a need for a gradual increase in antiepileptic drug dosing. Four days into the hospital admission, the patient developed a fever, shortness of breath, and hypoxemia. Aspiration pneumonia was assumed, leading to the initiation of piperacillin-tazobactam treatment.

The following day, owing to respiratory failure and altered mental status (Glasgow Coma Scale of 8), the patient was transferred to the intensive care unit (ICU), where an uncomplicated endotracheal intubation was performed. The patient clinically improved after 19 days, and an extubation attempt was made. However, it failed due to exhaustion and rapid desaturation. A subsequent intubation procedure was carried out without complications. After five days, a tracheostomy was performed due to difficulties in de-escalating mechanical ventilation, which was closed after 15 days.

Dysphagia was suspected because of the significant amount of secretions and sialorrhea, but confusion and poor collaboration delayed the performance of a swallowing test, and the patient was fed through a nasogastric tube.

It was only 24 days after extubation, while in the Internal Medicine nursery, that the patient, presenting persistent hoarseness and dysphagia for all food consistencies, underwent fibroscopy with a swallowing test and a meticulous neurological examination. The laryngoscopic examination was positive for right vocal cord paralysis, while the neurological examination revealed normal soft palate elevation with bilateral symmetric reflexes, a centered uvula, and deviation of the tongue to the right with ipsilateral atrophy. 

**Figure 1:**
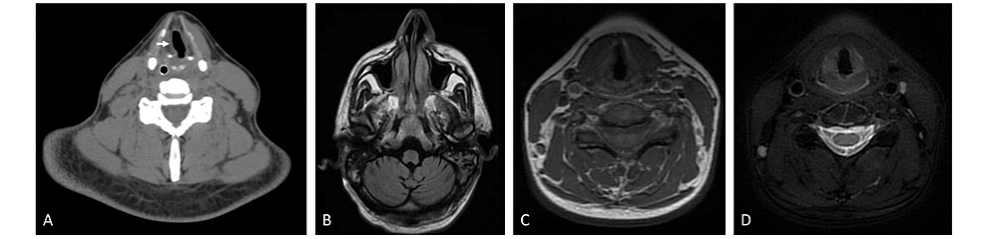
Imaging of the head and neck region. A. Neck CT revealing right vocal cord paralysis (arrow). B. Brain MRI did not reveal any brainstem lesions or extra-axial infratentorial masses, particularly located in the perimedullary cisterns. C, D. No cervical lesions were identified on neck MRI (C: T1 sequence; D: T2 sequence with fat suppression) CT: computed tomography; MRI: magnetic resonance imaging

Brain and neck magnetic resonance imaging (MRI) and computed tomography (CT) studies, including cervical angio-CT, showed no abnormalities besides right vocal cord paralysis on cervical CT (Figure 1). Cerebrospinal fluid analysis and autoimmune workup were also negative.

Tapia's syndrome was assumed due to peripheral paralysis of the hypoglossal nerve (XII) and the recurrent laryngeal branch of the vagal nerve (X), in the context of endotracheal oral intubation. The patient was started on prednisolone (1 mg/kg/d) with a slow reduction schedule over 12 weeks, and a prompt speech-language and swallowing rehabilitation plan was established with a multidisciplinary team. A percutaneous endoscopic gastrostomy (PEG) tube was placed.

Re-evaluation after three months showed no clear improvement. After six months, it was possible to remove the PEG tube, as the patient had recovered tongue motility with normal swallowing function. After 12 months, the voice was clear, and the patient revealed complete recovery.

## Discussion

Tapia's syndrome is a rare complication of airway manipulation, involving the combination of Xth and XIIth cranial nerve palsy [5]. The incidence of this clinical entity is likely underestimated, often due to clinician's lack of awareness.

To the best of our knowledge, only three documented cases of Tapia's syndrome exist in the literature following airway intubation for respiratory support in the context of medical illnesses in the ICU, without any surgery [4,6]. Nevertheless, a greater number of cases have been reported subsequent to surgical interventions performed under general anesthesia [2,3,5,7-14].

Various etiological mechanisms have been proposed for Tapia's syndrome, most frequently, but not exclusively, described as an iatrogenic condition [4]. Other causes include trauma, neoplasia, or dissection of the vertebral or carotid artery [7]. The majority of documented peripheral cases are attributed to orotracheal intubation [15], causing stretching and compression of nerves [5].

Anatomical anomalies, such as an enlarged hyoid bone, may predispose individuals to this injury [15], along with excessive mobilization of the head and neck during intubation [14,16]. Nerve damage may also be associated with rigid intubation and extubation maneuvers [3]. A neuropraxic reaction involving both nerves can result from the pressure exerted by a malpositioned or displaced tracheal tube [11], or from a hyperinflated cuff (pressure >30 cmH2O), potentially exacerbating the condition [2,15]. Depending on the duration of the injury, it is possible that the patient develops permanent neurotmesis of the nerve [4].

In the presented case of Tapia's syndrome, exposing the precise mechanisms underlying the nerve injury proves challenging. The utilization of two intubation techniques and the duration of endotracheal intubation and potential patient movements during partial seizures, which could dislocate the tube and exert pressure on the cuff at the intersection of the hypoglossal and recurrent laryngeal branch of the vagal nerve, are implicated as factors contributing to this neuropraxic injury.

The involvement of various specialties, including neurology, rehabilitation, and otolaryngology, is imperative for a comprehensive diagnosis, and the performance of airway fibroscopic laryngoscopy is, in our opinion, binding to ensure a reliable diagnosis [5].

The therapeutic approach to Tapia's syndrome is primarily supportive. Corticosteroids constitute the cornerstone of treatment [3], and some authors advocate for the additional use of vitamins (B1, B6, or B12 or combinations), though these medical treatments lack unequivocal scientific evidence support [4]. It is of utmost importance to establish a proper speech-language and swallowing rehabilitation program with adequate multidisciplinary support, in order to accomplish a faster and full recovery [5].

In consideration of the iatrogenic etiological mechanisms, physicians should adopt preventive measures, including (1) applying gentle pressure with the laryngoscope during intubation maneuvers; (2) employing a manometer to prevent endotracheal tube cuff hyperinflation; (3) implementing an effective endotracheal tube fixation system; and (4) exercising caution during head and neck maneuvers in both intubation and extubation procedures while avoiding neck hyperextension [2,4,8,9].

While most documented cases demonstrate full recovery within four to six months [3,5], the prolonged duration of endotracheal intubation for respiratory support may extend the recovery phase [7].

## Conclusions

After undergoing oral endotracheal intubation procedures, particularly in cases requiring prolonged respiratory support, the presence of persistent dysphagia and dysphonia, coupled with observable deviation or restriction of tongue movement, should prompt heightened suspicion of Tapia's syndrome. Recognizing the concurrent paralysis of both the hypoglossal nerve (XII) and the recurrent laryngeal branch of the vagal nerve (X) is pivotal for the comprehensive management of this condition.

The delayed diagnosis in the presented patient underscores the current lack of awareness and emphasizes the imperative for increased education regarding this rare yet clinically significant complication arising from airway manipulation. Hence, swift initiation of corticosteroid therapy, coupled with the implementation of a comprehensive speech-language and swallowing rehabilitation program, is paramount for achieving a prompt and complete recovery for the patient.
